# Tris{*N*-[(anthracen-9-yl)methyl­ene­amino]thio­ureato}cobalt(III) tetra­hydrate

**DOI:** 10.1107/S1600536808031425

**Published:** 2008-10-04

**Authors:** Jianying Zhao, Yu Zhang

**Affiliations:** a Department of Chemistry, Huaiyin Teachers College, Huai’an 223300, Jiangsu, People’s Republic of China

## Abstract

In the title complex, [Co(C_16_H_12_N_3_S)_3_]·4H_2_O, the central Co^III^ atom is in a distorted octa­hedral coordination environment. There are three *N*-[(anthracen-9-yl)­methyl­ene­amino]­thio­ureate ligands coordinated to the Co^III^ atom *via* three imine N and three thio­amide S atoms. The Co—S and Co—N bond distances are in expected ranges [2.2194 (8)—2.2545 (8) and 1.926 (2)—1.985 (2)Å, respectively]. The endocyclic S—Co—N bond angles in the five-membered chelate rings range from 82.91 (7) to 85.33 (7)°. The structure contains four water mol­ecules which are disordered over 12 sites and link the complex mol­ecules into a three-dimensional network through N—H⋯O, O—H⋯O, O—H⋯N, and O—H⋯S hydrogen bonds.

## Related literature

For related structures, see: Chandra *et al.* (2003 ▶); Funston *et al.* (2003 ▶); Casas *et al.* (2000 ▶); Rodriguez-Arguelles *et al.* (2004 ▶); Saha *et al.* (2003 ▶). For biological activities, see: He *et al.* (2003 ▶); Horton *et al.* (2003 ▶); Kabanos *et al.* (1992 ▶); Navarrete-Vazquez *et al.* (2001 ▶); Ozden *et al.*, 2005 ▶; Pawar *et al.* (2004 ▶).
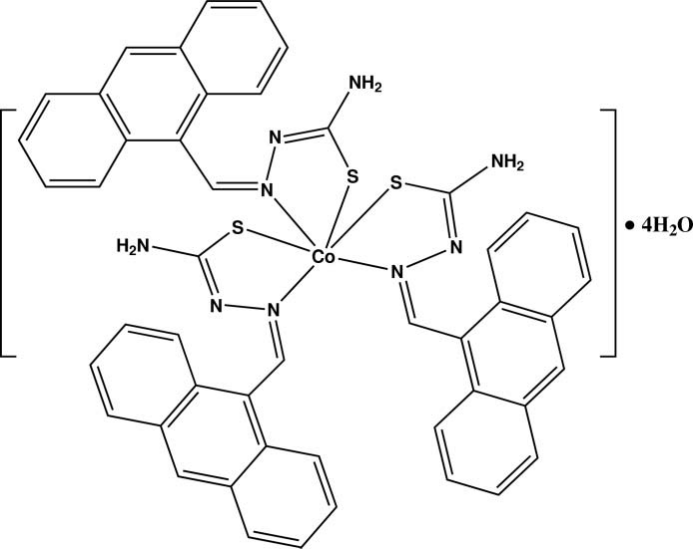


## Experimental

### 

#### Crystal data

[Co(C_16_H_12_N_3_S)_3_]·4H_2_O
*M* *_r_* = 966.03
Triclinic, 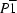

*a* = 9.8907 (19) Å
*b* = 17.073 (3) Å
*c* = 17.511 (4) Å
α = 91.315 (7)°
β = 99.920 (6)°
γ = 93.972 (6)°
*V* = 2903.9 (10) Å^3^
*Z* = 2
Mo *K*α radiation
μ = 0.45 mm^−1^
*T* = 291 (2) K
0.28 × 0.22 × 0.20 mm

#### Data collection

Bruker SMART APEX CCD diffractometer
Absorption correction: multi-scan (*SADABS*; Bruker, 2000 ▶) *T*_min_ = 0.886, *T*_max_ = 0.916
36568 measured reflections
11268 independent reflections
8244 reflections with *I* > 2σ(*I*)
*R*_int_ = 0.053

#### Refinement

*R*[*F* ^2^ > 2σ(*F* ^2^)] = 0.057
*wR*(*F* ^2^) = 0.132
*S* = 1.04
11268 reflections
723 parameters
H atoms treated by a mixture of independent and constrained refinement
Δρ_max_ = 0.90 e Å^−3^
Δρ_min_ = −0.92 e Å^−3^

### 

Data collection: *SMART* (Bruker, 2000 ▶); cell refinement: *SAINT* (Bruker, 2000 ▶); data reduction: *SAINT*; program(s) used to solve structure: *SHELXS97* (Sheldrick, 2008 ▶); program(s) used to refine structure: *SHELXL97* (Sheldrick, 2008 ▶); molecular graphics: *SHELXTL* (Sheldrick, 2008 ▶); software used to prepare material for publication: *SHELXTL*.

## Supplementary Material

Crystal structure: contains datablocks I, global. DOI: 10.1107/S1600536808031425/pv2097sup1.cif

Structure factors: contains datablocks I. DOI: 10.1107/S1600536808031425/pv2097Isup2.hkl

Additional supplementary materials:  crystallographic information; 3D view; checkCIF report

## Figures and Tables

**Table 1:** Hydrogen-bond geometry (Å, °)

*D*—H⋯*A*	*D*—H	H⋯*A*	*D*⋯*A*	*D*—H⋯*A*
N3—H3*A*⋯O10	0.85 (4)	2.36 (4)	3.130 (6)	151 (3)
N9—H9*B*⋯O7	0.85 (4)	2.38 (4)	3.162 (8)	153 (4)
N9—H9*B*⋯O8	0.85 (4)	2.47 (4)	3.002 (9)	122 (3)
N9—H9*A*⋯O11	0.85 (4)	2.24 (4)	2.957 (9)	142 (4)
O1—H1*X*⋯O6	0.82	1.59	2.263 (11)	137
O1—H1*X*⋯O11^i^	0.82	2.58	3.013 (11)	114
O7—H7*Y*⋯N3^ii^	0.82	2.62	3.158 (10)	124
O8—H8*Y*⋯S2^ii^	0.82	2.60	3.371 (10)	157
O9—H9*Y*⋯O11^iii^	0.82	2.59	3.300 (12)	146
O12—H12*X*⋯O11^iii^	0.82	2.25	3.003 (11)	153
O10—H10*X*⋯N9^iv^	0.82	2.26	3.033 (6)	157
O10—H10*Y*⋯N8^iv^	0.82	2.48	2.939 (6)	116
O11—H11*X*⋯S3	0.82	2.46	3.165 (8)	145
O11—H11*X*⋯N5	0.82	2.57	3.163 (9)	130
O11—H11*Y*⋯O1^v^	0.82	2.50	3.013 (11)	122
O12—H12*Y*⋯O9	0.82	2.06	2.494 (12)	113

Symmetry codes: (i) 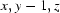; (ii) 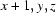; (iii) 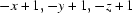; (iv) 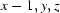; (v) 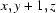.

**Fig. 1.**
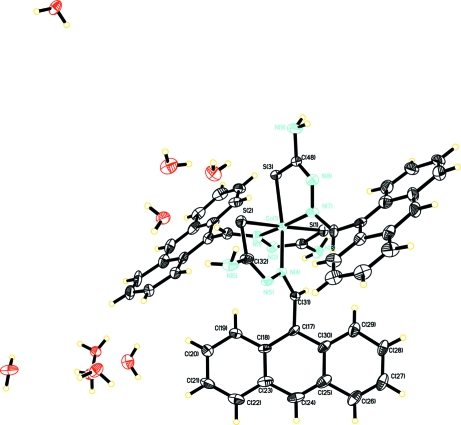
The molecular structure of (I), with atom labels and 30% probability displacement ellipsoids. H atoms have been omitted for clarity.

## supplementary crystallographic information

### Comment

The thiosemicarbazone derivatives and their transition metal complexes have received considerable attention because of their biological and pharmaceutical properties. These compounds have been previously investigated for their antifungal (Horton *et al.*, 2003), antibacterial (He *et al.*, 2003), antimicrobial (Pawar *et al.*, 2004), antiamoebic (Ozden *et al.*, 2005), antiparasitic (Navarrete-Vazquez *et al.*, 2001) and antitumor activities (Kabanos *et al.*, 1992). Although many thiosemicarbazones and their transition metal complexes have been studied (Rodriguez-Arguelles *et al.*, 2004), there is no information available on the structural characterization of anthracene thiosemicarbazone derivatives. The pharmacological or coordinative information on the anthracene thiosemicarbazone derivatives and their complexes are also unknown (Casas *et al.*, 2000). In this paper, we report the synthesis and crystal structure of tris(N-(9-anthracene) methylene-aniline thiourea)-cobalt(III) tetrahydrate complex, (I).

In the structure of (I), the central cobalt atom adopts a distorted octahedral geometry (Fig. 1). There are three ligands, N-(9-anthracene), methylene-aniline and thiourea which are coordinated to the cobalt atom *via* three imine nitrogen and three thioamide sulfur atoms. As expected, the sulfur and nitrogen atoms are in the *mer* conformation. The bond distances Co—S and Co—N, are in the expected ranges of 2.2194 (8)–2.2545 (8) and 1.926 (2)–1.985 (2) Å, respectively, which are in agreement with the literature values (Chandra *et al.*, 2003; Saha *et al.*, 2003). The bond lengths in the anthracene rings are typical and compareable to the values reported for the complex *trans*-dichloro-(6-(anthracen-9-ylmethyl) -1,4,8,11-tetraazacyclotetradecane)-cobalt(III) chloride pentahydrate (Funston *et al.*, 2003). The endocyclic bond angles S—Co—N in the five membered rings involving chelates range from 82.91 (7) to 85.33 (7)°. In the molecule there are three anthracene rings A (C1–C14), C (C17–C30), and E(C33–C46) and three five memberd chelating rings, B (Co1/N1/N2/C16/S1), D (Co1/N4/N5/C32/S2), and F (Co1/N7/N8/C48/S3). The dihedral angles between the mean-planes of the rings A and B, C and D, and E and F are 47.60 (4), 54.95 (6), and 51.51 (4)%, respectively.

There are four disordered water molecules in the structure which are located over twelve sites with partial occupancyies and take part in a bridging role, linking the complex molecules into a three-dimensional network through N—H···O, O—H···O, O—H···N, and O—H···S type hydrogen bonds (details are given in Table 1).

### Experimental

An ethanolic (25 ml) solution of Co(ClO_4_).6H_2_O (0.267 g, 0.10 mmol) was slowly added to N-(9-anthracene) methylene-aniline thiourea (0.837 g, 0.30 mmol) in ethanol (50 ml). The mixture was allowed to stand for 16 days at room temperature. Dark brown prismatic crystals suitable for X-ray analysis were obtained.

### Refinement

H atoms bonded to N were located in the difference map and were allowed to refine with distance restraints of N—H = 0.85 (4) Å, and *U*_iso_(H) = 1.2*U*_eq_(N). The H-atoms bonded to C-atoms were positioned geometrically and treated in a riding model, with C—H = 0.93 Å, and *U*_iso_(H) = 1.2 times *U*_eq_(C). The four molecules occupy 12 positions, the occupation factors for O1 to O12 were 0.390 (10), 0.301 (12), 0.309 (11), 0.400 (13), 0.304 (9), 0.297 (14), 0.392 (9), 0.300 (12), 0.308 (11), 0.396 (9), 0.299 (11) and 0.304 (9), respectively, which were refined as the free variables. The hydrogen atoms bonded to water molecules were positioned geometrically and refined using a riding model, with O—H = 0.82 Å and H—O—H angle at 104.5° and with *U*_iso_(H) = 1.2*U*_eq_(O). The highest electron density in the final difference map was located close to O11.

### Crystal data

**Table:**

[Co(C_16_H_12_N_3_S)_3_]·4H_2_O	*Z* = 2
*M**_r_* = 966.03	*F*(000) = 1004
Triclinic, *P*1	*D*_x_ = 1.105 Mg m^−^^3^
Hall symbol: -P 1	Mo *K*α radiation, λ = 0.71073 Å
*a* = 9.8907 (19) Å	Cell parameters from 5672 reflections
*b* = 17.073 (3) Å	θ = 2.1–25.1°
*c* = 17.511 (4) Å	µ = 0.45 mm^−^^1^
α = 91.315 (7)°	*T* = 291 K
β = 99.920 (6)°	Block, dark brown
γ = 93.972 (6)°	0.28 × 0.22 × 0.20 mm
*V* = 2903.9 (10) Å^3^	

### Data collection

**Table:**

Bruker SMART APEX CCD diffractometer	11268 independent reflections
Radiation source: fine-focus sealed tube	8244 reflections with *I* > 2σ(*I*)
graphite	*R*_int_ = 0.054
φ and ω scans	θ_max_ = 26.0°, θ_min_ = 1.2°
Absorption correction: multi-scan (SADABS; Bruker, 2000)	*h* = −11→12
*T*_min_ = 0.886, *T*_max_ = 0.916	*k* = −21→21
36568 measured reflections	*l* = −21→19

### Refinement

**Table:**

Refinement on *F*^2^	Primary atom site location: structure-invariant direct methods
Least-squares matrix: full	Secondary atom site location: difference Fourier map
*R*[*F*^2^ > 2σ(*F*^2^)] = 0.057	Hydrogen site location: inferred from neighbouring sites
*wR*(*F*^2^) = 0.132	H atoms treated by a mixture of independent and constrained refinement
*S* = 1.04	*w* = 1/[σ^2^(*F*_o_^2^) + (0.0616*P*)^2^ + 1.0884*P*] where *P* = (*F*_o_^2^ + 2*F*_c_^2^)/3
11268 reflections	(Δ/σ)_max_ < 0.001
723 parameters	Δρ_max_ = 0.90 e Å^−^^3^
0 restraints	Δρ_min_ = −0.92 e Å^−^^3^

### Special details

**Table:**

Geometry. All e.s.d.'s (except the e.s.d. in the dihedral angle between two l.s. planes) are estimated using the full covariance matrix. The cell e.s.d.'s are taken into account individually in the estimation of e.s.d.'s in distances, angles and torsion angles; correlations between e.s.d.'s in cell parameters are only used when they are defined by crystal symmetry. An approximate (isotropic) treatment of cell e.s.d.'s is used for estimating e.s.d.'s involving l.s. planes.Least-squares planes (*x*,*y*,*z* in crystal coordinates) and deviations from them (* indicates atom used to define plane)2.9231 (0.0039) *x* + 5.2249 (0.0137) *y* + 14.3798 (0.0073) *z* = 8.0721 (0.0041)* -0.0965 (0.0029) C1 * -0.0957 (0.0029) C2 * -0.0036 (0.0029) C3 * 0.0943 (0.0029) C4 * 0.1068 (0.0032) C5 * 0.0348 (0.0029) C6 * -0.0687 (0.0029) C7 * -0.0824 (0.0030) C8 * -0.0553 (0.0032) C9 * -0.0172 (0.0030) C10 * 0.0763 (0.0032) C11 * 0.0979 (0.0031) C12 * 0.0622 (0.0029) C13 * -0.0529 (0.0030) C14Rms deviation of fitted atoms = 0.0741- 4.9814 (0.0075) *x* + 4.3447 (0.0118) *y* + 15.7855 (0.0071) *z* = 7.5113 (0.0076)Angle to previous plane (with approximate e.s.d.) = 47.70 (0.05)* -0.0696 (0.0009) Co1 * 0.0932 (0.0014) N1 * -0.0516 (0.0018) N2 * -0.0286 (0.0017) C16 * 0.0566 (0.0011) S1Rms deviation of fitted atoms = 0.06362.8734 (0.0035) *x* + 5.6841 (0.0129) *y* + 14.2322 (0.0068) *z* = 7.3855 (0.0124)Angle to previous plane (with approximate e.s.d.) = 47.60 (0.05)* 0.0309 (0.0024) C17 * 0.0141 (0.0027) C18 * -0.0339 (0.0027) C19 * -0.0535 (0.0030) C20 * -0.0297 (0.0030) C21 * 0.0358 (0.0030) C22 * 0.0565 (0.0029) C23 * 0.0204 (0.0029) C24 * 0.0095 (0.0030) C25 * -0.0415 (0.0030) C26 * -0.0419 (0.0028) C27 * -0.0195 (0.0027) C28 * 0.0296 (0.0027) C29 * 0.0231 (0.0027) C30Rms deviation of fitted atoms = 0.0341- 9.1789 (0.0035) *x* + 2.1487 (0.0123) *y* - 3.5707 (0.0144) *z* = 0.8355 (0.0111)Angle to previous plane (with approximate e.s.d.) = 54.95 (0.06)* -0.0837 (0.0009) Co1 * 0.1056 (0.0013) N4 * -0.0460 (0.0017) N5 * -0.0487 (0.0018) C32 * 0.0728 (0.0012) S2Rms deviation of fitted atoms = 0.07486.3204 (0.0049) *x* + 12.0610 (0.0065) *y* + 0.3576 (0.0128) *z* = 7.7520 (0.0036)Angle to previous plane (with approximate e.s.d.) = 52.76 (0.06)* 0.0549 (0.0024) C33 * 0.0124 (0.0026) C34 * 0.0290 (0.0026) C35 * -0.0236 (0.0026) C36 * -0.0270 (0.0027) C37 * -0.0047 (0.0027) C38 * -0.0022 (0.0027) C39 * -0.0012 (0.0030) C40 * 0.0058 (0.0030) C41 * 0.0210 (0.0032) C42 * 0.0245 (0.0031) C43 * -0.0395 (0.0030) C44 * -0.0445 (0.0030) C45 * -0.0047 (0.0028) C46Rms deviation of fitted atoms = 0.0267- 0.9842 (0.0075) *x* + 16.5335 (0.0046) *y* - 4.5518 (0.0118) *z* = 10.6006 (0.0060)Angle to previous plane (with approximate e.s.d.) = 51.51 (0.05)* 0.1181 (0.0009) Co1 * -0.1461 (0.0013) N7 * 0.0569 (0.0016) N8 * 0.0834 (0.0015) C48 * -0.1122 (0.0010) S3Rms deviation of fitted atoms = 0.1078
Refinement. Refinement of *F*^2^ against ALL reflections. The weighted *R*-factor *wR* and goodness of fit *S* are based on *F*^2^, conventional *R*-factors *R* are based on *F*, with *F* set to zero for negative *F*^2^. The threshold expression of *F*^2^ > σ(*F*^2^) is used only for calculating *R*-factors(gt) *etc*. and is not relevant to the choice of reflections for refinement. *R*-factors based on *F*^2^ are statistically about twice as large as those based on *F*, and *R*- factors based on ALL data will be even larger.

### Fractional atomic coordinates and isotropic or equivalent isotropic displacement parameters (Å^2^)

**Table:**

	*x*	*y*	*z*	*U*_iso_*/*U*_eq_	Occ. (<1)
C1	−0.0004 (4)	0.49552 (19)	0.37467 (18)	0.0451 (8)	
C2	0.1076 (3)	0.44401 (18)	0.37149 (18)	0.0414 (7)	
C3	0.2263 (4)	0.4681 (2)	0.3450 (2)	0.0495 (8)	
H3	0.2351	0.5178	0.3249	0.059*	
C4	0.3333 (4)	0.4189 (2)	0.3479 (2)	0.0504 (8)	
H4	0.4131	0.4355	0.3296	0.060*	
C5	0.3203 (4)	0.3449 (2)	0.3783 (2)	0.0605 (10)	
H5	0.3914	0.3118	0.3799	0.073*	
C6	0.2032 (4)	0.3201 (2)	0.4062 (2)	0.0525 (9)	
H6	0.1958	0.2707	0.4270	0.063*	
C7	0.0951 (4)	0.3695 (2)	0.40299 (18)	0.0464 (8)	
C8	−0.0213 (4)	0.34682 (19)	0.4339 (2)	0.0501 (9)	
H8	−0.0299	0.2963	0.4521	0.060*	
C9	−0.1257 (4)	0.39649 (19)	0.4390 (2)	0.0507 (9)	
C10	−0.2356 (4)	0.3693 (2)	0.4739 (2)	0.0557 (10)	
H10	−0.2434	0.3185	0.4914	0.067*	
C11	−0.3298 (4)	0.4211 (2)	0.4807 (2)	0.0638 (11)	
H11	−0.4033	0.4055	0.5050	0.077*	
C12	−0.3229 (4)	0.4970 (2)	0.4532 (2)	0.0530 (9)	
H12	−0.3924	0.5298	0.4581	0.064*	
C13	−0.2153 (4)	0.5235 (2)	0.4192 (2)	0.0504 (8)	
H13	−0.2087	0.5748	0.4028	0.060*	
C14	−0.1148 (4)	0.47218 (18)	0.40945 (18)	0.0424 (7)	
C15	0.0225 (3)	0.57617 (17)	0.34798 (17)	0.0354 (6)	
H15	0.1031	0.6045	0.3716	0.043*	
C16	−0.2661 (3)	0.61425 (16)	0.22101 (18)	0.0339 (6)	
C17	−0.0070 (3)	0.93633 (15)	0.14856 (15)	0.0295 (6)	
C18	−0.1222 (3)	0.98154 (16)	0.15258 (17)	0.0344 (6)	
C19	−0.2345 (3)	0.95054 (17)	0.18427 (19)	0.0401 (7)	
H19	−0.2348	0.9001	0.2032	0.048*	
C20	−0.3452 (4)	0.9954 (2)	0.1873 (2)	0.0496 (8)	
H20	−0.4196	0.9750	0.2084	0.060*	
C21	−0.3457 (4)	1.07082 (19)	0.1590 (2)	0.0496 (8)	
H21	−0.4208	1.1004	0.1603	0.060*	
C22	−0.2347 (4)	1.1015 (2)	0.1289 (2)	0.0521 (9)	
H22	−0.2343	1.1522	0.1107	0.062*	
C23	−0.1224 (3)	1.05713 (18)	0.12542 (19)	0.0419 (7)	
C24	−0.0095 (4)	1.08603 (19)	0.08854 (19)	0.0464 (8)	
H24	−0.0109	1.1357	0.0678	0.056*	
C25	0.0994 (4)	1.0419 (2)	0.08340 (19)	0.0481 (8)	
C26	0.2076 (4)	1.0709 (2)	0.0464 (2)	0.0522 (9)	
H26	0.2044	1.1202	0.0250	0.063*	
C27	0.3174 (4)	1.0272 (2)	0.0417 (2)	0.0516 (9)	
H27	0.3883	1.0480	0.0179	0.062*	
C28	0.3252 (3)	0.95342 (19)	0.07113 (19)	0.0458 (8)	
H28	0.3994	0.9241	0.0667	0.055*	
C29	0.2176 (4)	0.92301 (19)	0.1084 (2)	0.0462 (8)	
H29	0.2224	0.8737	0.1298	0.055*	
C30	0.1043 (3)	0.96635 (17)	0.11354 (17)	0.0377 (7)	
C31	−0.0046 (3)	0.85530 (15)	0.17471 (16)	0.0285 (6)	
H31	−0.0042	0.8168	0.1363	0.034*	
C32	−0.0260 (3)	0.86221 (17)	0.36530 (18)	0.0384 (7)	
C33	0.0539 (3)	0.61759 (16)	0.04899 (16)	0.0329 (6)	
C34	−0.0004 (3)	0.64474 (17)	−0.02610 (18)	0.0388 (7)	
C35	−0.1012 (3)	0.69926 (18)	−0.0367 (2)	0.0460 (8)	
H35	−0.1346	0.7194	0.0056	0.055*	
C36	−0.1496 (4)	0.7224 (2)	−0.1095 (2)	0.0504 (9)	
H36	−0.2197	0.7565	−0.1163	0.060*	
C37	−0.0970 (4)	0.6965 (2)	−0.1755 (2)	0.0519 (9)	
H37	−0.1312	0.7145	−0.2243	0.062*	
C38	0.0059 (4)	0.6442 (2)	−0.1668 (2)	0.0516 (9)	
H38	0.0420	0.6276	−0.2096	0.062*	
C39	0.0548 (3)	0.61660 (19)	−0.09285 (19)	0.0450 (8)	
C40	0.1544 (4)	0.56412 (17)	−0.0809 (2)	0.0524 (9)	
H40	0.1896	0.5466	−0.1236	0.063*	
C41	0.2052 (3)	0.53602 (18)	−0.0105 (2)	0.0451 (8)	
C42	0.3102 (4)	0.4819 (2)	0.0004 (2)	0.0568 (9)	
H42	0.3459	0.4651	−0.0424	0.068*	
C43	0.3600 (4)	0.4540 (2)	0.0715 (2)	0.0538 (9)	
H43	0.4308	0.4204	0.0774	0.065*	
C44	0.3018 (4)	0.4773 (2)	0.1356 (2)	0.0519 (9)	
H44	0.3318	0.4568	0.1838	0.062*	
C45	0.2022 (4)	0.5293 (2)	0.1283 (2)	0.0524 (9)	
H45	0.1670	0.5439	0.1720	0.063*	
C46	0.1509 (3)	0.56158 (18)	0.05700 (19)	0.0427 (7)	
C47	−0.0038 (3)	0.64530 (16)	0.11451 (16)	0.0336 (6)	
H47	−0.0978	0.6342	0.1122	0.040*	
C48	0.2714 (3)	0.72902 (16)	0.24213 (17)	0.0329 (6)	
Co1	−0.01722 (4)	0.72094 (2)	0.26757 (2)	0.02906 (11)	
N1	−0.0574 (2)	0.61179 (13)	0.29523 (14)	0.0318 (5)	
N2	−0.1777 (2)	0.56985 (13)	0.25965 (13)	0.0324 (5)	
N3	−0.3917 (3)	0.58207 (18)	0.18756 (17)	0.0433 (6)	
H3B	−0.382 (4)	0.534 (2)	0.179 (2)	0.052*	
H3A	−0.440 (4)	0.598 (2)	0.147 (2)	0.052*	
N4	−0.0029 (2)	0.83041 (12)	0.24356 (13)	0.0254 (5)	
N5	0.0057 (2)	0.88954 (13)	0.29963 (14)	0.0320 (5)	
N6	−0.0234 (3)	0.91326 (19)	0.42362 (19)	0.0514 (8)	
H6B	−0.088 (4)	0.901 (2)	0.448 (2)	0.062*	
H6A	−0.033 (4)	0.959 (2)	0.406 (2)	0.062*	
N7	0.0635 (2)	0.68505 (13)	0.17780 (14)	0.0327 (5)	
N8	0.1979 (2)	0.70442 (13)	0.17449 (14)	0.0330 (5)	
N9	0.4071 (3)	0.7494 (2)	0.2452 (2)	0.0519 (8)	
H9B	0.454 (4)	0.721 (2)	0.278 (2)	0.062*	
H9A	0.425 (4)	0.798 (2)	0.259 (2)	0.062*	
O1	0.1493 (6)	0.0369 (3)	0.3188 (4)	0.048 (3)	0.390 (10)
H1X	0.2300	0.0392	0.3132	0.058*	0.39 (8)
H1Y	0.1521	0.0517	0.3640	0.058*	0.39 (8)
O2	0.2155 (9)	0.8786 (6)	0.6169 (6)	0.061 (4)	0.301 (12)
H2X	0.2050	0.8959	0.5732	0.073*	0.301 (9)
H2Y	0.2385	0.8339	0.6113	0.073*	0.301 (9)
O3	0.1224 (8)	0.7650 (6)	0.7284 (5)	0.055 (4)	0.309 (11)
H3X	0.1672	0.7269	0.7247	0.065*	0.309 (9)
H3Y	0.1548	0.7851	0.7712	0.065*	0.309 (8)
O4	0.4442 (8)	0.7059 (5)	0.7923 (5)	0.076 (4)	0.400 (13)
H4X	0.4462	0.6927	0.8372	0.091*	0.400 (11)
H4Y	0.5215	0.6993	0.7836	0.091*	0.400 (10)
O5	0.5254 (7)	0.7669 (6)	1.0002 (6)	0.055 (4)	0.304 (11)
H5X	0.5428	0.8096	1.0236	0.066*	0.304 (9)
H5Y	0.5234	0.7770	0.9545	0.066*	0.304 (9)
O6	0.3423 (14)	0.1055 (6)	0.2972 (7)	0.076 (6)	0.297 (14)
H6X	0.2957	0.1255	0.2604	0.091*	0.297 (12)
H6Y	0.4169	0.1014	0.2837	0.091*	0.297 (10)
O7	0.4935 (8)	0.6024 (4)	0.3438 (5)	0.065 (3)	0.392 (12)
H7X	0.4204	0.6001	0.3601	0.078*	0.392 (9)
H7Y	0.4804	0.5709	0.3068	0.078*	0.392 (10)
O8	0.6008 (10)	0.7804 (7)	0.3965 (6)	0.066 (5)	0.300 (12)
H8X	0.5613	0.7485	0.4210	0.079*	0.300 (10)
H8Y	0.6749	0.7624	0.3946	0.079*	0.300 (9)
O9	0.5014 (9)	0.1250 (5)	0.5405 (6)	0.057 (4)	0.308 (11)
H9X	0.5542	0.1629	0.5353	0.068*	0.308 (8)
H9Y	0.5461	0.0987	0.5730	0.068*	0.308 (9)
O10	−0.5834 (6)	0.6894 (3)	0.0820 (3)	0.040 (2)	0.396 (9)
H10X	−0.5752	0.7174	0.1215	0.048*	0.396 (7)
H10Y	−0.6562	0.6630	0.0808	0.048*	0.396 (7)
O11	0.3213 (9)	0.9052 (5)	0.2864 (5)	0.051 (3)	0.299 (11)
H11X	0.2617	0.8738	0.2975	0.061*	0.299 (8)
H11Y	0.3286	0.9408	0.3194	0.061*	0.299 (8)
O12	0.3964 (8)	0.0380 (4)	0.6305 (4)	0.041 (3)	0.304 (9)
H12X	0.4681	0.0659	0.6428	0.049*	0.304 (7)
H12Y	0.3782	0.0401	0.5831	0.049*	0.304 (7)
S1	−0.23532 (7)	0.71424 (4)	0.20858 (4)	0.03045 (16)	
S2	−0.06780 (7)	0.76419 (4)	0.37977 (4)	0.03041 (16)	
S3	0.20521 (7)	0.73627 (4)	0.32576 (4)	0.03020 (16)	

### Atomic displacement parameters (Å^2^)

**Table:**

	*U*^11^	*U*^22^	*U*^33^	*U*^12^	*U*^13^	*U*^23^
C1	0.055 (2)	0.0498 (18)	0.0292 (17)	0.0107 (15)	−0.0014 (14)	0.0085 (14)
C2	0.0452 (18)	0.0451 (16)	0.0315 (16)	−0.0018 (13)	0.0016 (14)	−0.0007 (13)
C3	0.054 (2)	0.056 (2)	0.040 (2)	0.0048 (16)	0.0127 (16)	−0.0105 (15)
C4	0.050 (2)	0.061 (2)	0.040 (2)	0.0086 (16)	0.0090 (16)	−0.0141 (16)
C5	0.061 (2)	0.068 (2)	0.052 (2)	−0.0215 (19)	0.0207 (19)	0.0004 (18)
C6	0.055 (2)	0.0536 (19)	0.043 (2)	−0.0176 (16)	0.0001 (16)	0.0068 (16)
C7	0.054 (2)	0.0527 (18)	0.0256 (16)	−0.0164 (16)	−0.0026 (14)	−0.0070 (14)
C8	0.054 (2)	0.0430 (17)	0.045 (2)	−0.0120 (15)	−0.0091 (16)	0.0158 (15)
C9	0.058 (2)	0.0423 (17)	0.044 (2)	−0.0014 (15)	−0.0107 (17)	0.0074 (14)
C10	0.045 (2)	0.0503 (19)	0.060 (2)	−0.0008 (15)	−0.0211 (18)	0.0073 (17)
C11	0.054 (2)	0.075 (3)	0.055 (2)	0.0200 (19)	−0.0152 (19)	0.004 (2)
C12	0.054 (2)	0.068 (2)	0.0380 (19)	0.0226 (17)	0.0055 (16)	0.0048 (16)
C13	0.047 (2)	0.070 (2)	0.0362 (19)	0.0193 (16)	0.0070 (15)	0.0017 (16)
C14	0.054 (2)	0.0458 (17)	0.0261 (16)	0.0120 (14)	0.0000 (14)	0.0045 (13)
C15	0.0332 (15)	0.0418 (15)	0.0308 (16)	0.0037 (12)	0.0032 (12)	0.0059 (12)
C16	0.0304 (15)	0.0316 (13)	0.0392 (17)	0.0005 (11)	0.0066 (12)	−0.0048 (12)
C17	0.0335 (14)	0.0313 (13)	0.0199 (13)	−0.0092 (11)	−0.0005 (11)	−0.0029 (10)
C18	0.0319 (15)	0.0376 (14)	0.0323 (16)	−0.0071 (11)	0.0070 (12)	−0.0089 (12)
C19	0.0446 (17)	0.0360 (15)	0.0425 (19)	0.0025 (12)	0.0156 (14)	0.0011 (13)
C20	0.0437 (19)	0.0543 (19)	0.051 (2)	0.0133 (15)	0.0053 (16)	−0.0027 (16)
C21	0.0474 (19)	0.0459 (18)	0.058 (2)	0.0074 (14)	0.0158 (17)	−0.0156 (16)
C22	0.0446 (19)	0.0510 (19)	0.060 (2)	−0.0004 (15)	0.0111 (17)	−0.0119 (17)
C23	0.0414 (17)	0.0415 (16)	0.0420 (18)	−0.0150 (13)	0.0141 (14)	−0.0114 (14)
C24	0.058 (2)	0.0477 (17)	0.0354 (18)	−0.0217 (15)	0.0259 (15)	−0.0075 (14)
C25	0.056 (2)	0.0495 (18)	0.0337 (18)	−0.0192 (16)	0.0042 (15)	−0.0006 (14)
C26	0.061 (2)	0.056 (2)	0.038 (2)	−0.0121 (17)	0.0085 (17)	0.0184 (16)
C27	0.058 (2)	0.0489 (18)	0.0406 (19)	−0.0233 (16)	−0.0036 (16)	0.0122 (15)
C28	0.0393 (17)	0.0466 (17)	0.0426 (19)	−0.0135 (13)	−0.0124 (14)	0.0100 (14)
C29	0.053 (2)	0.0393 (16)	0.0402 (19)	−0.0077 (14)	−0.0051 (15)	0.0033 (13)
C30	0.0427 (17)	0.0417 (15)	0.0246 (15)	−0.0119 (13)	0.0011 (13)	−0.0011 (12)
C31	0.0326 (14)	0.0260 (12)	0.0261 (15)	0.0078 (10)	0.0011 (11)	−0.0001 (10)
C32	0.0441 (17)	0.0351 (14)	0.0339 (17)	−0.0042 (12)	0.0049 (13)	−0.0089 (12)
C33	0.0406 (16)	0.0355 (14)	0.0200 (14)	−0.0050 (12)	0.0016 (12)	−0.0038 (11)
C34	0.0409 (17)	0.0396 (15)	0.0317 (16)	−0.0076 (12)	−0.0014 (13)	0.0037 (12)
C35	0.0468 (19)	0.0441 (17)	0.048 (2)	−0.0062 (14)	0.0135 (16)	0.0017 (15)
C36	0.0457 (19)	0.0495 (18)	0.053 (2)	−0.0158 (15)	0.0091 (16)	−0.0041 (16)
C37	0.054 (2)	0.0531 (19)	0.044 (2)	−0.0133 (16)	0.0030 (16)	0.0041 (16)
C38	0.064 (2)	0.0493 (18)	0.042 (2)	−0.0105 (16)	0.0150 (17)	0.0034 (15)
C39	0.0414 (18)	0.0444 (17)	0.0424 (19)	−0.0155 (14)	−0.0034 (15)	−0.0053 (14)
C40	0.079 (3)	0.0286 (14)	0.045 (2)	−0.0024 (15)	0.0009 (18)	−0.0004 (13)
C41	0.0434 (18)	0.0377 (15)	0.050 (2)	0.0052 (13)	−0.0036 (15)	−0.0020 (14)
C42	0.064 (2)	0.052 (2)	0.054 (2)	0.0149 (17)	0.0047 (19)	0.0014 (17)
C43	0.057 (2)	0.0507 (19)	0.049 (2)	0.0201 (16)	−0.0081 (17)	−0.0192 (16)
C44	0.049 (2)	0.0514 (19)	0.053 (2)	0.0117 (15)	0.0029 (17)	−0.0125 (16)
C45	0.055 (2)	0.055 (2)	0.047 (2)	0.0086 (16)	0.0036 (17)	0.0029 (16)
C46	0.0429 (17)	0.0398 (16)	0.0400 (18)	−0.0031 (13)	−0.0047 (14)	−0.0065 (13)
C47	0.0365 (15)	0.0388 (14)	0.0247 (15)	0.0169 (12)	−0.0030 (12)	0.0035 (11)
C48	0.0381 (16)	0.0325 (13)	0.0293 (15)	0.0083 (11)	0.0076 (12)	−0.0040 (11)
Co1	0.0298 (2)	0.02984 (19)	0.0273 (2)	0.00171 (14)	0.00459 (15)	−0.00025 (14)
N1	0.0262 (12)	0.0308 (11)	0.0387 (14)	0.0020 (9)	0.0062 (10)	0.0010 (10)
N2	0.0363 (13)	0.0337 (12)	0.0248 (12)	−0.0071 (10)	0.0023 (10)	0.0033 (9)
N3	0.0337 (14)	0.0530 (16)	0.0384 (16)	−0.0031 (12)	−0.0049 (12)	0.0016 (13)
N4	0.0235 (11)	0.0252 (10)	0.0251 (12)	−0.0057 (8)	0.0016 (9)	−0.0074 (9)
N5	0.0302 (12)	0.0365 (12)	0.0295 (13)	0.0019 (9)	0.0077 (10)	−0.0101 (10)
N6	0.0503 (17)	0.0517 (16)	0.0505 (19)	−0.0176 (14)	0.0152 (14)	−0.0189 (14)
N7	0.0421 (14)	0.0288 (11)	0.0273 (13)	−0.0043 (10)	0.0102 (10)	−0.0050 (9)
N8	0.0378 (13)	0.0291 (11)	0.0289 (13)	−0.0081 (9)	0.0017 (10)	−0.0025 (9)
N9	0.0481 (18)	0.0562 (18)	0.055 (2)	0.0013 (14)	0.0224 (15)	−0.0152 (15)
O1	0.038 (4)	0.066 (4)	0.037 (4)	−0.020 (3)	0.005 (3)	−0.002 (3)
O2	0.057 (6)	0.063 (6)	0.067 (7)	0.006 (4)	0.022 (5)	0.006 (4)
O3	0.035 (5)	0.089 (7)	0.038 (5)	0.002 (4)	0.003 (3)	−0.012 (4)
O4	0.058 (5)	0.090 (6)	0.074 (7)	−0.024 (4)	0.011 (4)	−0.013 (4)
O5	0.031 (4)	0.081 (7)	0.050 (6)	−0.013 (4)	0.006 (3)	0.010 (4)
O6	0.084 (9)	0.083 (8)	0.064 (8)	−0.025 (6)	0.038 (6)	−0.030 (6)
O7	0.054 (5)	0.057 (4)	0.077 (6)	−0.011 (3)	−0.001 (4)	0.005 (4)
O8	0.040 (6)	0.099 (8)	0.054 (6)	−0.003 (5)	0.005 (4)	−0.033 (5)
O9	0.052 (6)	0.049 (5)	0.068 (7)	0.003 (4)	0.009 (4)	−0.008 (4)
O10	0.034 (3)	0.049 (3)	0.035 (4)	0.005 (2)	0.004 (2)	−0.016 (2)
O11	0.064 (6)	0.046 (5)	0.036 (5)	−0.006 (4)	−0.003 (4)	0.008 (4)
O12	0.051 (5)	0.038 (4)	0.027 (4)	−0.015 (3)	−0.003 (3)	−0.001 (3)
S1	0.0306 (4)	0.0308 (3)	0.0294 (4)	0.0021 (3)	0.0041 (3)	0.0000 (3)
S2	0.0318 (4)	0.0314 (3)	0.0277 (4)	0.0015 (3)	0.0048 (3)	−0.0006 (3)
S3	0.0316 (4)	0.0317 (3)	0.0270 (4)	0.0016 (3)	0.0047 (3)	−0.0002 (3)

### Geometric parameters (Å, °)

**Table:**

C1—C14	1.413 (5)	C35—H35	0.9300
C1—C2	1.437 (5)	C36—C37	1.419 (5)
C1—C15	1.479 (4)	C36—H36	0.9300
C2—C3	1.376 (5)	C37—C38	1.392 (5)
C2—C7	1.404 (4)	C37—H37	0.9300
C3—C4	1.390 (5)	C38—C39	1.406 (5)
C3—H3	0.9300	C38—H38	0.9300
C4—C5	1.389 (5)	C39—C40	1.370 (5)
C4—H4	0.9300	C40—C41	1.360 (5)
C5—C6	1.376 (5)	C40—H40	0.9300
C5—H5	0.9300	C41—C42	1.429 (5)
C6—C7	1.401 (5)	C41—C46	1.450 (5)
C6—H6	0.9300	C42—C43	1.366 (5)
C7—C8	1.390 (5)	C42—H42	0.9300
C8—C9	1.394 (5)	C43—C44	1.408 (5)
C8—H8	0.9300	C43—H43	0.9300
C9—C10	1.393 (5)	C44—C45	1.363 (5)
C9—C14	1.407 (4)	C44—H44	0.9300
C10—C11	1.346 (5)	C45—C46	1.404 (5)
C10—H10	0.9300	C45—H45	0.9300
C11—C12	1.392 (5)	C47—N7	1.334 (4)
C11—H11	0.9300	C47—H47	0.9300
C12—C13	1.362 (5)	C48—N8	1.322 (4)
C12—H12	0.9300	C48—N9	1.354 (4)
C13—C14	1.398 (5)	C48—S3	1.710 (3)
C13—H13	0.9300	Co1—N4	1.926 (2)
C15—N1	1.300 (4)	Co1—N1	1.967 (2)
C15—H15	0.9300	Co1—N7	1.985 (2)
C16—N2	1.307 (4)	Co1—S1	2.2195 (9)
C16—N3	1.353 (4)	Co1—S2	2.2314 (9)
C16—S1	1.738 (3)	Co1—S3	2.2544 (9)
C17—C30	1.421 (4)	N1—N2	1.386 (3)
C17—C18	1.430 (4)	N3—O10	3.130 (6)
C17—C31	1.468 (3)	N3—H3B	0.85 (4)
C18—C23	1.386 (4)	N3—H3A	0.85 (4)
C18—C19	1.403 (4)	N4—N5	1.380 (3)
C19—C20	1.386 (4)	N6—H6B	0.85 (4)
C19—H19	0.9300	N6—H6A	0.85 (4)
C20—C21	1.390 (5)	N7—N8	1.359 (3)
C20—H20	0.9300	N9—O8	3.002 (9)
C21—C22	1.375 (5)	N9—O7	3.162 (8)
C21—H21	0.9300	N9—H9B	0.85 (4)
C22—C23	1.395 (5)	N9—H9A	0.85 (4)
C22—H22	0.9300	O1—H1X	0.8200
C23—C24	1.447 (4)	O1—H1Y	0.8200
C24—C25	1.370 (5)	O2—H2X	0.8200
C24—H24	0.9300	O2—H2Y	0.8200
C25—C30	1.406 (4)	O3—H3X	0.8200
C25—C26	1.410 (5)	O3—H3Y	0.8200
C26—C27	1.372 (5)	O4—H4X	0.8200
C26—H26	0.9300	O4—H4Y	0.8200
C27—C28	1.374 (4)	O5—H5X	0.8200
C27—H27	0.9300	O5—H5Y	0.8200
C28—C29	1.416 (5)	O6—H6X	0.8200
C28—H28	0.9300	O6—H6Y	0.8200
C29—C30	1.399 (5)	O7—H7X	0.8200
C29—H29	0.9300	O7—H7Y	0.8200
C31—N4	1.285 (3)	O8—H8X	0.8200
C31—H31	0.9300	O8—H8Y	0.8200
C32—N6	1.323 (4)	O9—H9X	0.8200
C32—N5	1.329 (4)	O9—H9Y	0.8200
C32—S2	1.731 (3)	O10—H10X	0.8200
C33—C46	1.393 (4)	O10—H10Y	0.8200
C33—C34	1.432 (4)	O11—H11X	0.8200
C33—C47	1.450 (4)	O11—H11Y	0.8200
C34—C35	1.403 (5)	O12—H12X	0.8200
C34—C39	1.458 (5)	O12—H12Y	0.8200
C35—C36	1.360 (5)		
			
C14—C1—C2	120.9 (3)	C35—C36—C37	122.5 (4)
C14—C1—C15	122.2 (3)	C35—C36—H36	118.7
C2—C1—C15	116.5 (3)	C37—C36—H36	118.7
C3—C2—C7	119.7 (3)	C38—C37—C36	119.7 (3)
C3—C2—C1	122.0 (3)	C38—C37—H37	120.2
C7—C2—C1	118.1 (3)	C36—C37—H37	120.2
C2—C3—C4	120.7 (3)	C37—C38—C39	119.4 (3)
C2—C3—H3	119.6	C37—C38—H38	120.3
C4—C3—H3	119.6	C39—C38—H38	120.3
C5—C4—C3	119.6 (4)	C40—C39—C38	122.1 (3)
C5—C4—H4	120.2	C40—C39—C34	118.3 (3)
C3—C4—H4	120.2	C38—C39—C34	119.6 (3)
C6—C5—C4	120.6 (4)	C41—C40—C39	124.2 (4)
C6—C5—H5	119.7	C41—C40—H40	117.9
C4—C5—H5	119.7	C39—C40—H40	117.9
C5—C6—C7	119.9 (3)	C40—C41—C42	123.0 (4)
C5—C6—H6	120.1	C40—C41—C46	119.0 (3)
C7—C6—H6	120.1	C42—C41—C46	118.0 (3)
C8—C7—C6	120.6 (3)	C43—C42—C41	122.3 (4)
C8—C7—C2	119.8 (3)	C43—C42—H42	118.8
C6—C7—C2	119.5 (3)	C41—C42—H42	118.8
C7—C8—C9	122.9 (3)	C42—C43—C44	118.6 (3)
C7—C8—H8	118.5	C42—C43—H43	120.7
C9—C8—H8	118.5	C44—C43—H43	120.7
C10—C9—C8	118.6 (3)	C45—C44—C43	121.3 (4)
C10—C9—C14	122.8 (3)	C45—C44—H44	119.4
C8—C9—C14	118.6 (3)	C43—C44—H44	119.4
C11—C10—C9	116.0 (4)	C44—C45—C46	122.2 (4)
C11—C10—H10	122.0	C44—C45—H45	118.9
C9—C10—H10	122.0	C46—C45—H45	118.9
C10—C11—C12	123.4 (4)	C33—C46—C45	123.1 (3)
C10—C11—H11	118.3	C33—C46—C41	119.3 (3)
C12—C11—H11	118.3	C45—C46—C41	117.5 (3)
C13—C12—C11	120.6 (4)	N7—C47—C33	127.0 (3)
C13—C12—H12	119.7	N7—C47—H47	116.5
C11—C12—H12	119.7	C33—C47—H47	116.5
C12—C13—C14	118.9 (3)	N8—C48—N9	118.0 (3)
C12—C13—H13	120.6	N8—C48—S3	123.8 (2)
C14—C13—H13	120.6	N9—C48—S3	118.3 (2)
C13—C14—C9	118.3 (3)	N4—Co1—N1	172.00 (9)
C13—C14—C1	122.0 (3)	N4—Co1—N7	94.66 (9)
C9—C14—C1	119.6 (3)	N1—Co1—N7	91.26 (10)
N1—C15—C1	127.2 (3)	N4—Co1—S1	88.56 (7)
N1—C15—H15	116.4	N1—Co1—S1	85.33 (7)
C1—C15—H15	116.4	N7—Co1—S1	97.49 (8)
N2—C16—N3	119.4 (3)	N4—Co1—S2	84.94 (7)
N2—C16—S1	124.8 (2)	N1—Co1—S2	90.23 (8)
N3—C16—S1	115.9 (2)	N7—Co1—S2	169.43 (7)
C30—C17—C18	120.9 (3)	S1—Co1—S2	93.06 (3)
C30—C17—C31	117.6 (3)	N4—Co1—S3	87.64 (6)
C18—C17—C31	121.3 (2)	N1—Co1—S3	98.44 (7)
C23—C18—C19	119.5 (3)	N7—Co1—S3	82.91 (7)
C23—C18—C17	119.7 (3)	S1—Co1—S3	176.20 (3)
C19—C18—C17	120.8 (3)	S2—Co1—S3	86.52 (3)
C20—C19—C18	119.7 (3)	C15—N1—N2	116.8 (2)
C20—C19—H19	120.1	C15—N1—Co1	123.06 (19)
C18—C19—H19	120.1	N2—N1—Co1	120.16 (17)
C19—C20—C21	120.5 (3)	C16—N2—N1	112.9 (2)
C19—C20—H20	119.8	C16—N3—O10	117.1 (2)
C21—C20—H20	119.8	C16—N3—H3B	106 (3)
C22—C21—C20	119.7 (3)	O10—N3—H3B	127 (3)
C22—C21—H21	120.2	C16—N3—H3A	126 (3)
C20—C21—H21	120.2	H3B—N3—H3A	106 (4)
C21—C22—C23	120.6 (3)	C31—N4—N5	113.9 (2)
C21—C22—H22	119.7	C31—N4—Co1	123.90 (18)
C23—C22—H22	119.7	N5—N4—Co1	122.16 (17)
C18—C23—C22	120.0 (3)	C32—N5—N4	111.6 (2)
C18—C23—C24	118.5 (3)	C32—N6—H6B	110 (3)
C22—C23—C24	121.4 (3)	C32—N6—H6A	110 (3)
C25—C24—C23	121.6 (3)	H6B—N6—H6A	109 (4)
C25—C24—H24	119.2	C47—N7—N8	113.1 (2)
C23—C24—H24	119.2	C47—N7—Co1	126.3 (2)
C24—C25—C30	120.7 (3)	N8—N7—Co1	120.45 (17)
C24—C25—C26	120.6 (3)	C48—N8—N7	113.3 (2)
C30—C25—C26	118.6 (3)	C48—N9—O8	121.9 (3)
C27—C26—C25	120.9 (3)	C48—N9—O7	92.1 (2)
C27—C26—H26	119.6	O8—N9—O7	62.7 (3)
C25—C26—H26	119.6	C48—N9—H9B	109 (3)
C26—C27—C28	121.7 (3)	O8—N9—H9B	45 (3)
C26—C27—H27	119.2	C48—N9—H9A	110 (3)
C28—C27—H27	119.2	O8—N9—H9A	65 (3)
C27—C28—C29	118.5 (3)	O7—N9—H9A	127 (3)
C27—C28—H28	120.7	H9B—N9—H9A	109 (4)
C29—C28—H28	120.7	H1X—O1—H1Y	104.5
C30—C29—C28	120.7 (3)	H2X—O2—H2Y	104.5
C30—C29—H29	119.6	H3X—O3—H3Y	104.5
C28—C29—H29	119.6	H4X—O4—H4Y	104.5
C29—C30—C25	119.6 (3)	H5X—O5—H5Y	104.5
C29—C30—C17	121.9 (3)	H6X—O6—H6Y	104.5
C25—C30—C17	118.5 (3)	N9—O7—H7X	90.9
N4—C31—C17	128.7 (2)	N9—O7—H7Y	95.5
N4—C31—H31	115.7	H7X—O7—H7Y	104.5
C17—C31—H31	115.7	N9—O8—H8X	95.9
N6—C32—N5	117.7 (3)	N9—O8—H8Y	110.5
N6—C32—S2	118.1 (3)	H8X—O8—H8Y	104.5
N5—C32—S2	124.2 (2)	H9X—O9—H9Y	104.5
C46—C33—C34	120.4 (3)	N3—O10—H10X	84.2
C46—C33—C47	121.6 (3)	N3—O10—H10Y	98.0
C34—C33—C47	117.8 (3)	H10X—O10—H10Y	104.5
C35—C34—C33	122.0 (3)	H11X—O11—H11Y	104.5
C35—C34—C39	119.4 (3)	H12X—O12—H12Y	104.5
C33—C34—C39	118.6 (3)	C16—S1—Co1	95.29 (10)
C36—C35—C34	119.3 (3)	C32—S2—Co1	94.97 (11)
C36—C35—H35	120.3	C48—S3—Co1	95.59 (10)
C34—C35—H35	120.3		

### Hydrogen-bond geometry (Å, °)

**Table:**

*D*—H···*A*	*D*—H	H···*A*	*D*···*A*	*D*—H···*A*
N3—H3A···O10	0.85 (4)	2.36 (4)	3.130 (6)	151 (3)
N9—H9B···O7	0.85 (4)	2.38 (4)	3.162 (8)	153 (4)
N9—H9B···O8	0.85 (4)	2.47 (4)	3.002 (9)	122 (3)
N9—H9A···O11	0.85 (4)	2.24 (4)	2.957 (9)	142 (4)
O1—H1X···O6	0.82	1.59	2.263 (11)	137
O1—H1X···O11^i^	0.82	2.58	3.013 (11)	114
O7—H7Y···N3^ii^	0.82	2.62	3.158 (10)	124
O8—H8Y···S2^ii^	0.82	2.60	3.371 (10)	157
O9—H9Y···O11^iii^	0.82	2.59	3.300 (12)	146
O12—H12X···O11^iii^	0.82	2.25	3.003 (11)	153
O10—H10X···N9^iv^	0.82	2.26	3.033 (6)	157
O10—H10Y···N8^iv^	0.82	2.48	2.939 (6)	116
O11—H11X···S3	0.82	2.46	3.165 (8)	145
O11—H11X···N5	0.82	2.57	3.163 (9)	130
O11—H11Y···O1^v^	0.82	2.50	3.013 (11)	122
O12—H12Y···O9	0.82	2.06	2.494 (12)	113

Symmetry codes: (i) *x*, *y*−1, *z*; (ii) *x*+1, *y*, *z*; (iii) −*x*+1, −*y*+1, −*z*+1; (iv) *x*−1, *y*, *z*; (v) *x*, *y*+1, *z*.
